# Epidemiology of patient safety incidents in a long-term rehabilitative hospital in KwaZulu-Natal, South Africa (April 2011 to March 2016)

**DOI:** 10.4102/curationis.v44i1.2151

**Published:** 2021-05-18

**Authors:** Phe Mgobozi, Ozayr H. Mahomed

**Affiliations:** 1 Department of Public Health Medicine, University of KwaZulu-Natal, Durban, South Africa

**Keywords:** patient safety, falls, infection control, quality, pressure sores, rehabilitative hospital

## Abstract

**Background:**

Patient safety is a key priority of the National Department of Health. Despite the publication of legislation and other measures to address patient safety incidents (PSIs) there are a paucity of studies relating to patient safety at the different levels of hospitals.

**Objectives:**

To determine the epidemiology (incidence, nature and root causes) of PSIs at a long-term rehabilitative hospital between April 2011 and March 2016.

**Method:**

Data were collected through a review and analysis of routinely collected hospital information on patient records and from the PSI register, as well as minutes of adverse health events meetings, quality assurance reports and patient complaints register.

**Results:**

A total or 4.12 PSIs per 10 000 inpatient days were reported. Approximately 52% of the adverse health events occurred in females with most of the adverse health events occurring in the 50–59 years category: 96% being reported during the day and 33% within the shift change. Pressure ulcers, falls, injury, hospital acquired infections and medication error were the most commonly reported PSIs. Patient factors were listed as the most common root cause for the PSIs.

**Conclusion:**

The study shows a low reporting rate of PSIs whilst showing a diverse pattern of PSIs over a period of 5 years. There is a need for active change management in order to establish a blame-free culture and learning environment to improve reporting of PSI. A comprehensive quality improvement intervention addressing patients, their families and staff is essential to minimise PSI and its consequences.

## Introduction

Patient safety incidents (PSIs) continue to be a problem in healthcare delivery with patient harm being ranked the 14th leading cause of morbidity and mortality globally (Jha et al. 2013). Estimates indicate that approximately 42.7 million PSIs occur amongst an estimated 421 million hospitalisations in the world annually (Jha et al. 2013). Results from developed countries indicate that the rates of PSI were at least 8% of all hospital admissions with more than 50% judged to be preventable and deaths of between 0.5% and 2.0% of patients in hospital are associated with PSI (De Vries et al. 2008). A retrospective review of patients’ hospital records across eight lower-middle income countries (Egypt, Jordan, Kenya, Morocco, Tunisia, Sudan, South Africa and Yemen) estimated the frequency of patient harm at 8.2% with a range of 2.5% to 18.4% per country. Of these events, 83% were judged to be preventable, whilst about 30% were associated with the death of the patient (Wilson et al. 2012).

A study conducted amongst gynaecology patients admitted at King Edward Hospital, Durban showed that PSI occurred in 11.7% of admissions and 52% were deemed avoidable (Matsaseng & Moodley 2005). The majority of PSIs were minor (disability lasting less than 6 months). Mortality accounted for 17.7% of PSI and 2.1% of all admissions (Matsaseng & Moodley 2005). A recent study conducted at the same institutions using voluntary reporting of PSI showed that the overall reporting rate of PSIs were 3.8 incidents per 1000 patients between 2011 and 2014 (Mahomed & Kalonji 2017). There were five main PSIs identified. Perinatal incidents (25%) were associated with patients < 31 years of age and were more likely to occur at night (*p* < 0.05). Absconding (18%) was associated with male patients from acute admission and general wards (*p* < 0.05). Patient falls (11%) were associated with older patients and were more likely to occur in medical disciplines (*p* < 0.05). Pressure ulcers (7%) were strongly associated with a length of stay of longer than 5 days (*p* < 0.05). Obstetric organ injury (6%) was mostly attributed to patient factors (*p* < 0.05) (Mahomed & Kalonji 2017).

Patient harm does not only impose a significant burden on patients and their loved ones, it also generates a considerable strain on health system finances. Patient safety incidents necessitate the use of additional resources and increased levels of care. The financial burden across all categories of PSI occurring in hospitals varies from 1.3% to 32% of public hospital spending (Andel et al. 2012). The economic burden of PSI in Canada in 2009–2010 was $1 071 983 610.00 ($1.1 billion), including $396 633 936.00 ($397 million) for preventable PSI (Etchells et al. 2017). Estimates indicate that PSIs cost Irish hospitals more than EUR 194 million a year, equivalent to 4% of the healthcare acute services’ budget (Rafter et al. 2017). A retrospective review of 7926 patient records in 21 Dutch hospitals in 2004 estimated at a total of Euro 355m for all PSI and Euro 161m for preventable adverse events in 2004. The total number of hospital admissions in which a preventable adverse event occurred was 30 000 (2.3% of all admissions) and more than 300 000 (over 3% of all bed days) bed days were attributable to preventable adverse events (Hoonhout et al. 2009). A study conducted a retrospective examination of medical records in 13 public hospitals in New Zealand showed that PSI identified 850 PSI, which cost an average of $NZ 10 264.00 (New Zealand dollar) per patient. Patient safety incidents are estimated to cost the medical system $NZ 870m, of which $NZ 590m went towards treating preventable PSI. The results suggest that up to 30% of public hospital expenditure goes towards treating an adverse event (Brown et al. 2002).

In the public health sector of South Africa there is limited data on the cost of PSI. However, as a proxy estimate the scale of the medico-legal claims can be used to approximate the cost. By the end of the 2016–2017 fiscal year, government faced contingent liabilities – the cost if all claims were successful – of R56.1bn. That equates to almost a third of the R170.9bn consolidated health budget for 2016–2017 (Kahn 2018).

The Eastern Cape health department’s contingent liabilities rose from R16.8bn in 2016–2017 to R24.3bn in 2017–2018. In Gauteng, the figure rose from R17.8bn to R22bn over the period, whilst in the Limpopo’s province the amount doubled from R2.1bn to R4.35bn (Kahn 2018).

Patient safety is a key priority of the National Department of Health. The *National Health Act* Amended (Act No. 12 of 2013) mandates that health establishments develop an approach to identification and monitoring of PSIs and near misses. The Act stipulates that health establishments must maintain a surveillance system to collect, categorise and analyse adverse health events (Presidency of South Africa 2013). In addition, health establishments are mandated to report on PSIs to the relevant authorities and to determine the root causes thereof. Corrective actions must be implemented within specified time frames according to severity. Health establishments are expected to inform users of the protocols to be followed in the event of PSI. Finally, information related to each PSI must result in changes to processes, systems and behaviours to minimise or militate against the PSI (Presidency of South Africa 2013).

Despite the publication of legislation and other measures to address PSIs there is a paucity of PSI-specific information available in South Africa.

### Conceptual framework

**FIGURE 1:**
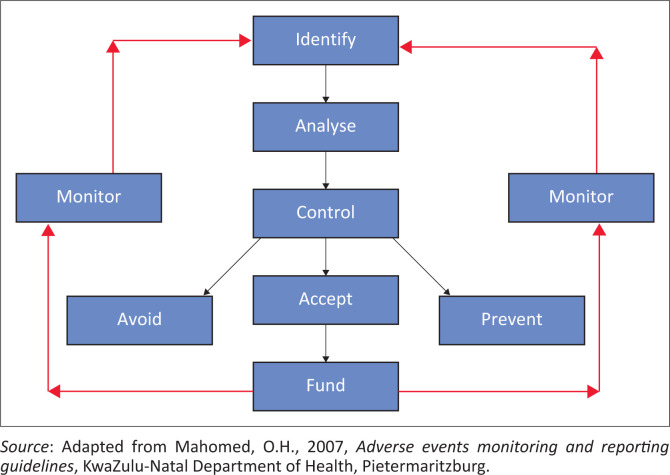
Risk management cycle for adverse events monitoring.

In 2007, the KwaZulu-Natal Department of Health developed guidelines for creating a standardised methodology of determining the nature, incidence, causes and reporting of adverse health events and designing of standard operating procedures for preventative actions (Mahomed 2007). These guidelines were superseded by the National Policy for Patient Safety Incident Reporting and Learning (National Department of Health 2016). The underlying approach towards adverse event monitoring is that of risk management. It includes development of risk management culture, standardisation of terminology, identification of PSI, root cause analysis of any PSI and classification of AHEs and appropriate remedial actions developed (Figure 1).

### Aim and objectives

The aim of this study was to determine the incidence, nature and root causes of PSIs at a long-term step-down hospital between April 2011 and March 2016. The specific objectives of the study were:

- to measure the quantity of formal incident reports of PSI from April 2011 to April 2016
- to describe the socio-demographic profile of patients who experienced PSI
- to determine the nature and root causes of PSIs.

## Research methods and design

### Study design

A retrospective cohort study was conducted of all patients admitted between April 2011 and March 2016.

### Setting

The hospital is a specialised chronic care facility providing care for patients with chronic non-communicable diseases and those that require physical rehabilitation. The hospital is categorised as a specialised step-down convalescent and step-down rehabilitation facility. The facility has 212 authorised beds and currently 175 usable beds and 5 wards (3 female wards and 2 male wards) and an outpatient department for chronic conditions with a head count ranging between 490 and 550 outpatients on the register per month. Common outpatients conditions consulted were stroke, traumatic injuries (quadriplegic, paraplegic and traumatic amputees) non-communicable complications of cardiac, neurological and orthopaedic diseases. The catchment population for the facility is the entire KwaZulu-Natal province. Services provided are rehabilitation services, which include occupational, physiotherapy and speech therapy, social services, pharmacy and outpatient clinic for repeat chronic medicines. The institution has commenced physiotherapy and occupational therapy for outpatients on a small-scale to support clients who are on the waiting list for admission.

### Study population and sampling strategy

The study population included all patients who were hospitalised at Hillcrest hospital between 01 April 2011 and 31 March 2016. All the records of inpatients who had reported adverse events during the period of study were reviewed. The entire population was included and no sampling was carried out.

### Data Collection

Data were collected from the adverse health events register, PSIs file, as well as minutes of adverse health event meetings. Some adverse events were identified from the quality assurance reports and complaints register, where complaints relating to adverse events were also identified.

Data collection was carried out in two phases: The first phase was to review all charts of patients who appeared on the adverse health events Register. The patient’s records and additional evidence of the adverse events were then retrieved. The adverse events reports that included detailed information on incident type, nature and description of incident and the root cause analysis and measurement of severity were reviewed. A data extraction sheet was used to collect the data.

The monitoring and evaluation personnel and the nursing manager were responsible for reviewing the event characteristics, which includes type of incident, nature, root cause, measures of consequence or impact, measures of likelihood, risk score and actions taken to reduce the frequency of adverse health events. All three professionals were experienced people who are exposed to auditing and hospital management.

### Reliability

The adverse health events were reported by the primary carer immediately after the incident occurred or was identified. The incident reporting forms were completed by healthcare provider with a full description of the incident. The doctor in charge of the patient is responsible for filling the doctors’ observations section. The operational manager or senior professional nurse of the unit then verifies a completed form before it is submitted to the quality assurance manager. The adverse health event is then investigated and discussed at the Hospital Patient Safety Incident Committee, Clinical Risk and Complaints Committee.

### Data management and analysis

All the data from the paper-based records were extracted on a Data Collection sheet. The information from the data collection sheet was then entered on Microsoft Excel. The data were exported to Software for Statistics and Data Science for analysis. Descriptive statistical analysis was used to describe baseline characteristics such as age, gender education level. The mean and median was used for continuous variables and frequencies and proportions were used for categorical variables. Frequency tables were generated for categorical variables showing frequency and percentage of socio-demographic characteristics and nature of adverse health events.

### Ethical considerations

The study was approved by the Biomedical Research and Ethics Committee (BREC) of the University of KwaZulu-Natal (BE096/17), the KwaZulu-Natal Provincial Health Research Ethics Committee (PHREC) (169/17).

## Results

### Incidence of adverse health events

During the period under study there were a total of 831 admissions translating to 264 515 inpatient days. A total of 110 PSIs fulfilled the inclusion criteria representing 13% of patients were admitted to the hospital or 4.12 PSIs per 10 000 inpatient days. The incidence of PSI fluctuated over the years reaching a peak of 40.9% of admissions (45) or 8.7 per 10 000 inpatient days in 2013/2014 and declined to 15.4% of admissions (17) or 3.4 per 10 000 inpatient days in 2014/2015 and 16.3% of admissions (18) or 3.7 per 10 000 inpatient days in 2015/2016 (Table 1).

**TABLE 1:** The annual and overall reported rates of adverse health events at Hillcrest hospital from April 2011 to March 2016.

Year of reporting	Number of admissions	Number of incident reports	Percentage of admissions with an incident reported (%)	Annual inpatient days	Incident reporting rate (per 10 000 inpatient days)
2011/12	172	11	10.0	58 419	1.8
2012/13	178	19	3.3	56 957	3.3
2013/14	162	45	40.9	51 576	8.7
2014/15	160	17	15.4	49 289	3.4
2015/16	159	18	16.3	48 274	3.7

**Total**	**831**	**110**	**13.0**	**264 515**	**4.1**

### Patient demographics

**FIGURE 2:**
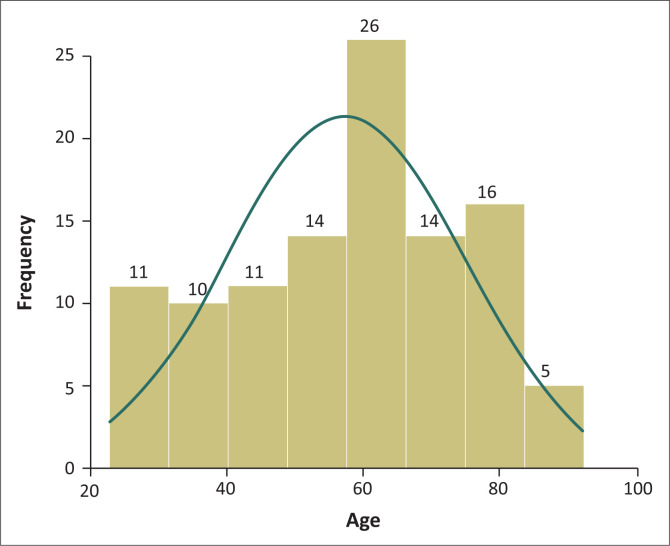
Age distribution of patients with adverse health events at Hillcrest hospital from April 2011 to March 2016.

A total of 52% (58/110) of the PSI reports involved female patients and 48% male patients. The mean age (*N* = 59) was 57.2 years, (standard deviation [SD] 17.03) with median age of 60 years (interquartile range [IQR] 20–80 years) denoting a positively skewed age distribution. Most PSI reported (23.8%; 26/110) were in the 50–59 years category and 25.6% (28/110) were in the 70 years and above category (Figure 2).

### Nature of reported patient safety incidents

Over the 5-year period pressure ulcers 32.0% (6.6 per 100 000 inpatient days), falls 24.0%, (4.2 per 10 000 inpatient days), injury 29.0% (2.5 per 10 000 inpatient days), hospital acquired infections 11.0% (2.4 per 10 000 inpatient days) and medication error (6/110) 5.4% (1.0 per 10.000 inpatient days) were the most common reported PSI (Table 2).

**TABLE 2:** Rate of reporting of the most prevalent adverse health events at Hillcrest hospital (per 100 000 inpatient days) from April 2011 to March 2016.

Year of reporting	Annual inpatient days	Rate of pressure ulcers reported		Rate of falls reported		Rate of injury reported		Rate of hospital acquired infection reported		Rate of medication errors reported	
		Number	Rate per 100 000 patient-days	Number	Rate per 100 000 patient-days	Number	Rate per 100 000 patient-days	Number	Rate per 100 000 patient-days	Number	Rate per 100 000 patient days
2011/2012	58 419	6	91.0	1	1.7	2	3.2	0	00.0	1	1.70
2012/2013	56 957	17	3.0	10	17.5	7	3.0	4	7.0	1	1.75
2013/2014	51 576	3	6.0	8	4.4	3	1.5	4	2.0	3	5.80
2014/2015	49 289	6	1.0	3	6.0	4	8.0	1	2.0	0	0.00
2015/2016	48 274	3	6.0	4	8.2	2	4.0	3	6.2	1	2.00
Overall reporting rate over 3 years	593 589	39	6.6	25	4.2	15	2.5	14	2.4	6	1.00

### Seriousness of the patient safety incidents

The majority (54.0%; *n* = 59) of PSIs were recorded as moderate followed by minor 37.0% (*n* = 40), 2.8% (*n* = 3) were classified as PSI where the patient suffered permanent disability or death.

### Root causes of patient safety incidents

The majority of the PSIs were directly related to the patient’s clinical condition. The most common diagnostic conditions of patients with reported PSIs of pressure ulcers and patient falls were neurovascular condition 63% (*n* = 69), mental health 15% (*n* = 17), epilepsy 8% (*n* = 9) and head injury 4% (*n* = 4). A total of 22% (25/110) of patients were exposed to PSI whilst under the effect of sedation medication. Staffing factors were the most common contributing factors, although 76% (*n* = 84) of PSI were reported where staffing was adequate compared with the 11% (*n* = 26), which was affected by a low nurse patient ratio. The main reasons for staff factors contributing to PSI included inadequate allocation of staff, inexperienced staff, inadequate communication and suboptimal adherence to protocols for patient safety. System factors and inadequate equipment were minor contributing factors.

## Discussion

The findings of this study indicate that 13% of inpatients suffered adverse health events, with pressure ulcers, falls, injury and hospital acquired infections and medication error being the most commonly reported PSI. The given findings, based on voluntary reporting are consistent with findings from many international studies (Schade et al. 2008). Voluntary reporting collects only a small proportion of adverse events (around 1% – 10%), which are not representative of all adverse events. The majority of these adverse events reported are falls, pressure ulcers and drug-related events whereas these constitute only 26% of adverse events detected by case note review (Sari et al. 2007a). In comparison, the Harvard Medical Practice Study’s using retrospective chart review studies estimated that between 4% and 17% of hospital admissions are associated with an adverse event and a significant proportion of these (one- to two-thirds) are preventable (Brennan et al. 1991), whilst research in hospitals in London and Scotland demonstrated adverse event rates of approximately 10% (Sari et al. 2007b). A systematic review of eight chart review studies (from the United States, Australia, the United Kingdom, New Zealand and Canada) found a median overall incidence of adverse events of 9.2% (of which approximately 43.0% were preventable), with over half being surgically related (40.0%) or drug (15.0%) related (De Vries et al. 2008).

However, it should be observed that there were inconsistencies in reporting of PSI in this study as some PSIs were only discovered on the minutes of meetings such as mortality and morbidity minutes and Infection Prevention and Control minutes, which were not reported as PSI to the hospital. Furthermore, the low incidence of reported adverse health events at the current site of 4.1 per 10 000 inpatient days is contrary to expectations as long-term rehabilitation required for patients admitted to this facility could be attributed to chronic long-term nature rehabilitation with patient length of stay ranging from 48 274 to 58 419 inpatient days.

Although an Adverse Health Event Monitoring and Reporting system was implemented in KwaZulu-Natal from 2008 onwards there was no enforcement of the system. Facilities only commenced actively reporting and monitoring adverse health events once official incident reporting documentation was introduced in 2011 through the Ideal Clinic system (National Department of Health 2011). Together with the introduction of the Patient Incident Reporting from, the province re-enforced the Adverse Events monitoring and implementation guidelines and this could explain the higher percentage of PSI reported in 2013/2014.

The majority of PSI in this study were in the older age group (> 60 years). Although mainly older age patients were admitted and this could account for higher number of adverse health events in the older age, a study from a large hospital in the National Health Service in the United Kingdom demonstrated that each year of life increased the risk of an adverse event by 28%, after adjustment for length of stay (Sari, Cracknell & Sheldon 2008). Longer length of stay, comorbidities, postural hypotension and mental health condition such as depression and delirium increase the risk of PSI in older patients because of their increased requirement for care. Adamuz et al.’s 2020. A systematic review using data from eight studies conducted in the United States, United Kingdom, New Zealand, Canada and Australia showed that most PSI resulted in little or no disability, a significant minority (median 14%) caused permanent disability (7%) or death (7%) (De Vries et al. 2008). In this study only 2.8% of the adverse health events of patients were serious where the patients suffered permanent disability or death. The most serious AHEs reported were aspiration pneumonia and dehydration, which led to death of patients. Pressure ulcers, body injuries related to fall, healthcare associated infections and medication error were the most common PSI at the facility.

The profile of patients, structure and processes of clinical care contribute to adverse health events (Braithwaite et al. 2011). In this study patient factors followed by staff factors were the main contributing factors to adverse health events. The majority of patients who experienced adverse health events in this study were admitted with neurological or mental health-related conditions. A systematic review of literature has indicated that loss of functional ability was independently associated with adverse events (Long et al. 2013; Thomas & Brennan 2000).

### Study limitations

The availability of good quality data and information are critical for making the right evidence-based decisions, whether for research, policy development or to ensure accountability to senior management and the general public. Data were collected routinely for clinical purposes and not for research purposes hence gaps were identified. Quality of the source data were at times compromised because of illegibility of handwriting and entries being made by different personnel with different understanding and skills thus affecting data collection. However, an attempt was made to address this by collecting data from multiple sources and use of a standardised tool.

The data were collected from a single hospital – a chronic step-down institution. Hence, the adverse events are contextual and are likely to be different from those at acute care hospital.

## Conclusion

This study was able to find a diverse pattern of adverse events in terms of type, incidence and contributing causes in a long-term chronic hospital that are similar to those identified in previous research, despite the low reporting of voluntary adverse health events. The low reporting could be an indication of some reluctance on the part of service providers to report incidents. Patient factors (length of stay, age, comorbidities, mental status) were the main contributing factors to the adverse health events with a low percentage because of staff and institutional factors.

### Recommendations

A comprehensive quality improvement intervention addressing patients, their families and staff is essential to minimise PSI and its consequences. Furthermore, there is a need for active change management in order to establish a blame-free culture and learning environment to improve reporting of PSI.
